# Complete response following trastuzumab-deruxtecan in a man with HER2-overexpressing metastatic, poorly differentiated scrotal carcinoma

**DOI:** 10.1093/oncolo/oyaf398

**Published:** 2025-12-02

**Authors:** Mia Hofstad, Jie Zheng, Isamu Tachibana, Tian Zhang

**Affiliations:** Simmons Comprehensive Cancer Center, Division of Hematology and Oncology, Department of Internal Medicine, University of Texas Southwestern Medical Center, Dallas, TX 75390, United States; Simmons Comprehensive Cancer Center, Division of Hematology and Oncology, Department of Internal Medicine, University of Texas Southwestern Medical Center, Dallas, TX 75390, United States; Department of Urology, University of Texas Southwestern Medical Center, Dallas, TX 75390, United States; Simmons Comprehensive Cancer Center, Division of Hematology and Oncology, Department of Internal Medicine, University of Texas Southwestern Medical Center, Dallas, TX 75390, United States

**Keywords:** human epidermal growth factor receptor 2, HER2, antibody-drug conjugates, trastuzumab-deruxtecan, scrotal cancer

## Abstract

Overexpression of human epidermal growth factor receptor 2 (HER2) has been implicated as a molecular driver of numerous solid tumor subtypes. An antibody drug conjugate (ADC) directed to HER2, trastuzumab-deruxtecan, recently gained tumor-agnostic FDA approval for the treatment of advanced HER2-positive (IHC 3+) solid tumors. Here, we present a patient with HER2-positive invasive poorly differentiated scrotal carcinoma treated with trastuzumab-deruxtecan. The treatment resulted in a complete response, with a tolerable side effect profile and ongoing treatment-free survival. Our case adds to the literature suggesting clinical benefit of the use of trastuzumab-deruxtecan in HER2-positive tumors, and underscores the importance of molecular testing for patients with rare tumor subtypes.

## Background

Human epidermal growth factor receptor 2 (HER2) is a receptor tyrosine kinase whose overexpression is linked to increased tumorigenesis due to unrestrained cellular proliferation.^1^^,^^2^ HER2 overexpression has been noted in a variety of solid tumors, notably breast and gastric cancers, but also colorectal, lung, and urothelial cancers, among others.^3^

Recently, clinical investigation has focused on the potential use of the antibody-drug conjugate (ADC) trastuzumab-deruxtecan (T-DXd) to target HER2-driven cancers. Indeed, results of the DESTINY-PanTumor02 trial, which investigated the efficacy of T-DXd in patients with HER2-positive solid tumors, demonstrated clinically meaningful progression-free survival and overall survival in patients with unresectable HER2-positive cancers.^2^ This clinical trial, combined with results of the DESTINY-Lung01 and DESTINY-CRC02 trials, led to accelerated FDA approval of T-DXd for metastatic or unresectable HER2-positive tumors.^4^

Here, we present the case of a 72-year-old man who presented with metastatic, poorly differentiated scrotal carcinoma found to overexpress HER2. Based on the molecular profile of his tumor, T-DXd treatment was initiated instead of cisplatin-based chemotherapy, resulting in complete response and continued treatment-free survival at the time of report. Our case adds to the literature suggesting clinical benefit of using T-DXd as effective treatment for HER2-positive tumors, and underscores the importance of molecular testing for patients with rare tumor types.

## Case

A 72-year-old man presented in January 2024 with a new, painless, slowly growing scrotal mass and associated overlying skin lesion. Past medical history was significant for treated prostate adenocarcinoma with undetectable PSA and a history of prior injury to the scrotal area. A biopsy of the overlying skin was obtained, with pathology demonstrating a poorly differentiated carcinoma with a nested pattern. Initial immunohistochemistry (IHC) staining was positive for GATA3, CK7, and CEA and negative for NKX3.1, PSA, CK20, TTF1, and BerEP4.

The mass was subsequently resected with negative surgical margins in February 2024. Pathology demonstrated a poorly differentiated primary scrotal carcinoma with lymphovascular invasion. Molecular testing demonstrated potentially actionable mutations in *PIK3CA* (p.E545K missense variant gain of function) and *ERBB2* (HER2) amplification. Subsequent IHC confirmed HER2 overexpression (3+).

**Figure 1.**
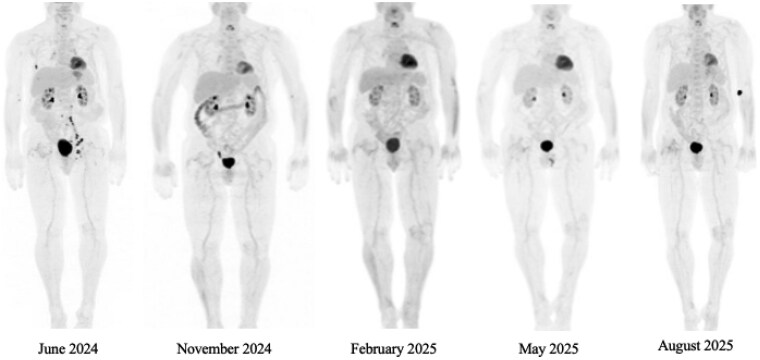
F18-fluorodeoxyglucose PET CT imaging. Patient FDG PET CT imaging over time. From left to right, coronal images of FDG PET (June 2024) before treatment initiation demonstrates increased FDG avidity, suggesting metastasis in the liver, left proximal femur, right rib, and peritoneum. FDG PET CT in November 2024 after 7 cycles of therapy demonstrates resolution of prior metastatic disease and FDG uptake. FDG PET CTs in February, May, and August 2025 maintained complete response.

In May 2024, follow-up CT demonstrated retroperitoneal and left iliac chain lymphadenopathy suspicious for metastatic disease. Confirmatory FDG PET CT imaging demonstrated metastases to the liver, left proximal femur, rib, and peritoneum (Figure 1). Discussion of systemic treatment options included TIP (paclitaxel, ifosfamide, and cisplatin) versus T-DXd. T-DXd was recommended, due to its recent approval for tumor-agnostic HER2 overexpressing tumors.^4^

The patient received his first infusion of T-DXd in July 2024. He required a dose-reduction after three cycles due to nausea and fatigue, but otherwise tolerated treatments well. After seven cycles in November 2024, his FDG PET CT demonstrated no evidence of continued tumor burden (Figure 1). After thorough discussion of radiographic response and his reluctance for any potential treatment-related toxicities, T-DXd was subsequently held. He is currently on ongoing surveillance with whole body FDG PET CTs every three months. As of his last follow-up in August 2025, he has maintained a complete response with no evidence of disease. His case demonstrates an exceptional response to T-DXd in a rare cancer subtype with HER2-positivity, and gives credence to its usage for HER2 overexpressing solid tumors. Importantly, this case highlights a role for molecular testing in all patients with rare solid tumors to identify targetable molecular drivers.

## Discussion

HER2 is a receptor tyrosine kinase that functions to promote downstream cellular survival and proliferation. In cancer, HER2 alterations commonly occur via HER2 protein overexpression or gene amplification leading to uncontrolled downstream signaling. HER2 overexpression/amplification is a common tumorigenic factor in a number of cancer subtypes, with one study demonstrating HER2 overexpression in 2.7% of all solid tumors.^5^

The ADC at the center of this case report, T-DXd, is a targeted therapy comprised of a HER2 directed monoclonal antibody linked to a topoisomerase I inhibitor payload.^2^ Recently, the combined results of three clinical trials (DESTINY-Lung01, DESTINY-CRC02, and DESTINY-PanTumor02) led to its FDA approval for adult patients with unresectable or metastatic HER2-positive (IHC 3+) solid tumors who have received prior systemic treatment or have no satisfactory alternative treatment options.^4^ The DESTINY-Lung01 trial enrolled patients with HER2-positive pretreated metastatic non-small cell lung cancer, while the DESTINY-CRC02 trial enrolled patients with HER2-positive metastatic colorectal cancer refractory to prior standard therapy or for whom prior therapy was contraindicated. Both trials demonstrated benefit in patients with HER2-positive cancers.^6^^,^^7^ The DESTINY-PanTumor02 study expanded on these findings by exploring the use of T-DXd in patients with HER2-overexpressing locally advanced or metastatic solid tumors, irrespective of the cell type of origin. Clinically meaningful progression-free survival and overall survival were demonstrated in patients with unresectable HER2-positive solid tumors, with a greater effect seen in patients with high expression of HER2 (IHC 3+).^2^

The broad applicability of DESTINY-PanTumor02 provided a compelling clinical rationale for using T-DXd for our patient, who presented with a metastatic HER2-high (3+) positive tumor, and for whom the alternative chemotherapy regimen (cisplatin/ifosfamide/paclitaxel) would have mandated inpatient administration and monitoring for ifosfamide-related neurotoxicity and hemorrhagic cystitis. Our patient’s exceptional response to T-DXd is consistent with results from the aforementioned trials, but importantly adds to the literature, as we demonstrate efficacy in a rare tumor subtype not included in the T-DXd trials. Interestingly, our patient demonstrated continued treatment-free survival despite treatment cessation, which is not well addressed in prospective trials, and further investigation is warranted to determine if this outcome is common at the population level.

Questions remain about the efficacy of T-DXd in tumors with intermediate expression of HER2 (IHC 1+ or 2+). Indeed, in the DESTINY-PanTumor02 study, a significant subset of patients with IHC 2+ HER2 expression responded to T-DXd therapy, and studies have demonstrated a significant increase in overall survival and progression-free survival in HER2-low breast cancers.^2^^,^^8^ Another HER2 directed ADC, disitimab vedotin, has also shown efficacy for HER2 expressing but non-amplified (IHC 1+/2+) urothelial cancers.^9^ Further investigation is warranted to assess the effectiveness of T-DXd use in this clinical context for rare tumors.

## Funding

Funding support for this article was provided by the Cancer Prevention and Research Institute of Texas (RR210079).

## Conflicts of interest

M.H., J.Z., and I.T. have no conflicts of interest. T.Z. has PI/research funding from Novartis, Merck, Janssen, Astra Zeneca, Pfizer, Astellas, Eli Lilly, Tempus, ALX Oncology, Janux, OncoC4, Exelixis, Bayer, and Kura Oncology and is on the Advisory Board/Consults for Merck, Exelixis, Sanofi-Aventis, Janssen, Astra Zeneca, Pfizer, Amgen, BMS, Eisai, Aveo, Eli Lilly, Bayer, Gilead, Novartis, EMD Serono, Dendreon, Xencor, Astellas, MJH Associates, Vaniam, Aptitude Health, PeerView, and Aravive.
